# Neutrally Buoyant Particle Migration in Poiseuille Flow Driven by Pulsatile Velocity

**DOI:** 10.3390/mi12091075

**Published:** 2021-09-06

**Authors:** Lizhong Huang, Jiayou Du, Zefei Zhu

**Affiliations:** School of Mechanical Engineering, Hangzhou Dianzi University, Hangzhou 310018, China; ricky@hdu.edu.cn (L.H.); abc@hdu.edu.cn (J.D.)

**Keywords:** lattice Boltzmann method, inertial migration, Poiseuille flow, pulsatile velocity

## Abstract

A neutrally buoyant circular particle migration in two-dimensional (2D) Poiseuille channel flow driven by pulsatile velocity is numerical studied by using immersed boundary-lattice Boltzmann method (IB-LBM). The effects of Reynolds number (25≤Re≤200) and blockage ratio (0.15≤k≤0.40) on particle migration driven by pulsatile and non-pulsatile velocity are all numerically investigated for comparison. The results show that, different from non-pulsatile cases, the particle will migrate back to channel centerline with underdamped oscillation during the time period with zero-velocity in pulsatile cases. The maximum lateral travel distance of the particle in one cycle of periodic motion will increase with increasing Re, while k has little impact. The quasi frequency of such oscillation has almost no business with Re and k. Moreover, Re plays an essential role in the damping ratio. Pulsatile flow field is ubiquitous in aorta and other arteries. This article is conducive to understanding nanoparticle migration in those arteries.

## 1. Introduction

Particle two-phase flow is a very complex problem, which ubiquitously exists in nature, industry, hemodynamics, such as the formation and movement of sand dunes, haze (PM2.5), ventilation dusting system, spread of virus (COVID-19), inertial microfluidics, drug delivery in blood, etc. Numerous researches have revealed the behavior of the particles in fluid flow depends on Reynolds number ($Re=U_{m}H/\nu$, where $U_{m}$ is the maximum inlet velocity, H is the channel width, ν is the fluid kinematic viscosity) and blockage ratio ($k=D_{p}/H$, donates the ratio of particle diameter $D_{p}$ and the channel width), whether or not the particles are neutrally buoyant [1,2,3,4,5,6]. The density of the neutrally buoyant particles is the same as the suspension fluid, which means the particles will suspend in the fluid. 

Segré and Silberberg [7] first discovered experimentally neutrally buoyant spherical particles would migrate to a radial equilibrium position in a pipe flow and form the Segré and Silberberg (SS) annulus, which is known as SS effect. This phenomenon prompted a lot of correlation research to reveal the underlying mechanism. Ho and Leal [8] theoretically studied particle migration in two-dimensional (2D) Poiseuille flow. Asmolov [9] interpreted that the particles migration was due to the effect of inertial lift by using the matched asymptotic expansions method. The results indicated the wall induced inertial lift became significant in the thin layers near the channel wall, and such lift could be neglected when the particles are far away from the wall. Matas et al. [10] found that the particles would move closer to the circular tube wall as Re increased and revealed additional inner annulus when Re was greater than 600. Moreover, if Re exceeded 700, the particles in the inner annulus accounted for the majority. Matas et al. [11] also utilized the matched asymptotic expansions method to calculate the lateral force in the pipe geometry (used to be in the plane geometry), but they did not find the second zero lateral force intersection point which indicates the inner annulus. Thus, they concluded the inner annulus was most likely due to finite-size effect. Hood et al. [12] calculated the lateral forces by a perturbation analysis. Morita et al. [13] predicted that all particles in the inner annulus would return to the SS annulus according to their experimental results when Re is less than 1000 and the tube is long enough. Due to their equipment limitations, the tube length was only 500 times the particle diameter. Then, Nakayama et al. [14] increased the length of the tube to 1000 times of the particle diameter, and the results showed that three regimes were eventually formed: only the SS annulus, only the inner annulus or those two annuli exist at the same time. The transition between these three regimes was determined by the critical Re which decreases with increasing of k.

Besides the aforementioned theoretical and experimental methods, computational fluid dynamics (CFD) simulation has become a powerful tool in analyzing particle-fluid interaction. Feng et al. [15] adopted finite-element method to investigate a circular particle migration in Poiseuille flow. Their simulations agreed qualitatively with the results of perturbation theories and pertinent experiments. By using LBM [16,17,18,19], the same problem was studied, and the SS effect was reconstructed. Shao et al. [20] found the inner annulus for elevated Re by using the fictitious domain method. Abbas et al. [21] mentioned that the equilibrium position (TEP) depends exclusively on Re and k. Recently, inertial microfluidics can precisely separate particles with or without extra external force field by realizing SS effect [22,23,24,25,26,27,28].

All the research works mentioned above are based on a non-pulsatile flow field, but in the artery, the blood pumped by the heart behaves as a pulsatile flow field. To the best of our knowledge, there are no articles related to particle migration in the pulsatile flow field. 

Cancer is one of the leading causes of death in the world; nanoparticles are widely used for cancer therapy. Ideally, the therapeutic nanoparticles system should be able to deliver drugs just to the tumor and have no severe side effects on the body [29]. However, nanoparticle movement in non-pulsatile blood flow field is quite different from that in the pulsatile blood flow field. 

In this study, we perform a series of CFD simulations to investigate the migration of one neutrally buoyant circular particle in 2D Poiseuille flow driven by pulsatile velocity. The influence of Re and k on the particle migration is analyzed in detail. The difference between particle migration driven in pulsatile and non-pulsatile velocity is illustrated. Engineering precision therapeutic nanoparticles system in artery [30] can be attainable by understanding the particle migration in pulsatile blood flow field. Furthermore, it can help to optimize therapeutic nanoparticles system for achieving precise medical against cancer near arteries.

The organization of this article is as follows. The numerical method, boundary conditions, and problem description are introduced in Section 2. In Section 3, we simulated TEPs of one particle at several specific parameters, and our computational code is validated by comparing with the published paper. Afterwards, the simulation results are presented and discussed in Section 4. Finally, conclusions are provided in Section 5.

## 2. Method and Problem

LBM is widely used to simulate particle migration, turbulence, flow in porous media, multiphase flow, non-Newtonian rheology, and so on [31,32,33,34,35,36,37,38,39,40,41,42,43]. Because of its advantages in efficiency, easy to code, and parallel run, LBM has become a very popular CFD numerical tool.

### 2.1. Lattice Boltzmann Method

**Figure 1:**
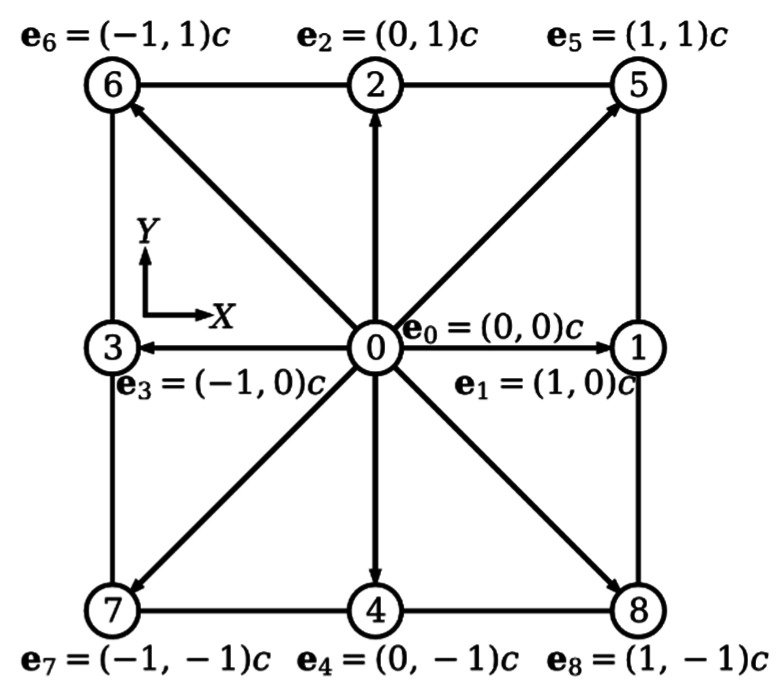
D2Q9 Cartesian lattice and discrete velocities.

In this work, the single-relaxation time (SRT) lattice Bhatnagar–Gross–Krook (LBGK) Boltzmann method is adopted to solve particles migration in incompressible viscous flow [44]:

(1) $$f_{i}\left(x+e_{i}\Delta t,t+\Delta t\right)=f_{i}\left(x,t\right)-\frac{1}{\tau }\left[f_{i}\left(x,t\right)-f_{i}^{\left(eq\right)}\left(x,t\right)\right],$$

where $f_{i}\left(x,t\right)$ is the distribution function at space coordinate x=(X,Y) and time t in the ith direction; $f_{i}^{\left(eq\right)}\left(x,t\right)$ is the correspond equilibrium distribution function; τ is the SRT; Δt is the time step; the discrete velocities $e_{i}$ of 2D nine-velocity (D2Q9) model are shown in Figure 1; c=Δx/Δt is the lattice velocity; Δx is the lattice spacing. For coding conveniently, both the time step and the lattice spacing are set to be equal to 1, which result in Δt=Δx=c=1.

The equilibrium distribution function can be calculated by [44]:

(2) $$f_{i}^{\left(eq\right)}\left(x,t\right)=\omega_{i}\rho_{f}\left[1+\frac{3e_{i}\cdot u}{c^{2}}+\frac{4.5{\left(e_{i}\cdot u\right)}^{2}}{c^{4}}-\frac{1.5u^{2}}{c^{2}}\right],$$

where $\omega_{i}$ is the weight factor with $\omega_{0}=4/9$, $\omega_{1\sim 4}=1/9$ and $\omega_{5\sim 8}=1/36$, $\rho_{f}$ is the fluid density and u is fluid velocity which can be determined by:

(3) $$\rho_{f}=\sum^{\;}f_{i}\left(x,t\right),\;u=\frac{1}{\rho_{f}}\sum^{\;}e_{i}f_{i}\left(x,t\right).$$

For low Mach number, the Navier–Stokes equations can be derived from the lattice Boltzmann equation by utilizing Chapman–Enskog expansion [45].

### 2.2. Improved Bounce-Back Scheme

In the LBM simulations, improved bounce-back scheme is of particular importance, and it allows to implement no-slip boundary condition on the surface of moving particle [46], which will be explained briefly below.

**Figure 2:**
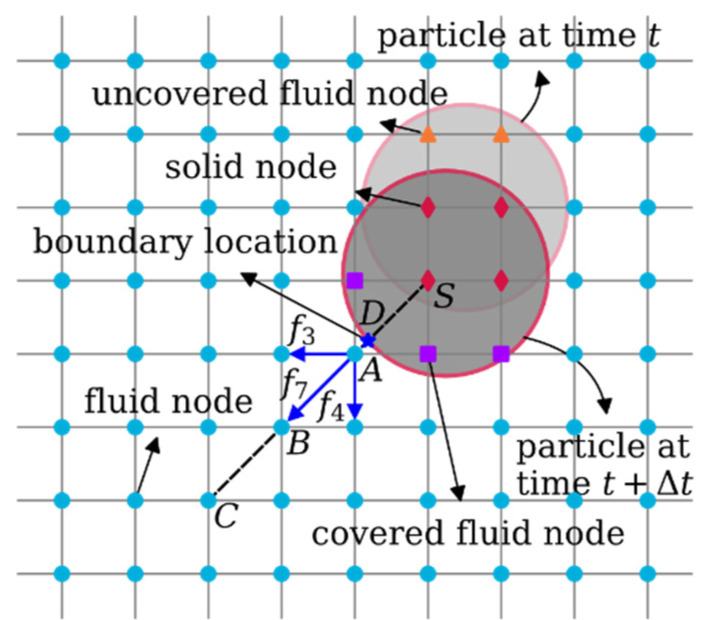
Improved bounce-back scheme boundary conditions. Lighter gray circle represents the location of particle at time t and darker gray circle at time t+∆t, which result in two orange triangles uncover to fluid node, three purple squares cover to solid node and four red thin diamonds remain solid node. The other sky-blue circles denote the fluid nodes. The distribution function $f_{7}$ can be determined by the information of node S, D, A, B, C.

As shown in Figure 2, the sky-blue circles represent the fluid nodes, and the red thin diamonds donate the solid nodes. The orange triangles indicate uncovered fluid nodes means that those nodes are located inside the particle at time t and will locate outside the particle when the particle travels after one lattice time step. Similarly, the purple squares donate covered fluid nodes represent that those nodes will change from fluid nodes to solid nodes after the particle moves. 

Take fluid node A as an example, after the streaming step, three unknown distribution functions ($f_{3}$, $f_{7}$ and $f_{4}$ denoted by three blue arrows shown in Figure 2) need to be determined by applying improved bounce-back scheme. Therefore, the distribution function $f_{7}$ can be calculated by:

(4) $$f_{7}\left(A\right)=\{\begin{array}{ll} q\left(1+2q\right)f_{5}\left(S\right)+\left(1-4q^{2}\right)f_{5}\left(A\right)-q\left(1-2q\right)f_{5}\left(B\right)-2\omega_{5}\rho_{f}\frac{e_{5}\cdot u_{D}}{c_{s}^{2}},\; & q<\frac{1}{2},\\ \frac{1}{q\left(2q+1\right)}f_{5}\left(S\right)+\frac{2q-1}{q}f_{7}\left(B\right)-\frac{2q-1}{2q+1}f_{7}\left(C\right)-\frac{2\omega_{5}\rho_{f}}{q\left(2q+1\right)}\frac{e_{5}\cdot u_{D}}{c_{s}^{2}},\; & q⩾\frac{1}{2}, \end{array}$$

where S is the nearest solid node along $e_{5}$ direction; B and C are the two nearest fluid nodes along $e_{7}$ direction; the blue star D denotes the boundary location which is the intersection point of particle boundary and line AS, meanwhile, q can be determined by q=|AD|/|AS|; $u_{D}$ is the velocity of the boundary location D; $c_{s}=c/\sqrt{3}$ is the speed of sound. The other unknown distribution functions of fluid nodes around particle boundary can be solved in the similar method.

### 2.3. Force, Torque, and Particle Motion

Let us continue to take the boundary location D as an example. As shown in Figure 2, the hydrodynamic force and torque acting on the solid particle migrating in fluid can be integrated by adopting momentum exchange algorithm [46,47] as follows:

(5) $$\begin{array}{l} F^{\left(h\right)}\left(x+qe_{5},t\right)=e_{5}\left[f_{5}\left(x+e_{5},t\right)+f_{7}\left(x,t\right)\right],\\ T^{\left(h\right)}\left(x+qe_{5},t\right)=\left(x+qe_{5}-x_{p}\right)\times F^{\left(h\right)}\left(x,t\right), \end{array}$$

where $x_{p}$ is the position of the particle.

When the particle is moving in the lattice grid from time t to t+∆t, as shown in Figure 2, the additional force and torque due to uncovered fluid node and covered fluid node exerted on the particle can be computed by [48]:

(6) $$\begin{array}{l} F^{\left(c\right)}\left(x,t\right)=\rho_{f}\left(x,t\right)u\left(x,t\right),\\ \begin{array}{l} T^{\left(c\right)}\left(x,t\right)=\left(x-x_{p}\right)\times F^{\left(c\right)}\left(x,t\right),\\ F^{\left(u\right)}\left(x,t\right)=-\rho_{f}\left(x,t\right)u\left(x,t\right), \end{array}\\ T^{\left(u\right)}\left(x,t\right)=\left(x-x_{p}\right)\times F^{\left(u\right)}\left(x,t\right). \end{array}$$

Moreover, in order to avoid unphysical overlapping between the particle and the channel wall, the extra lubrication force model is needed [49]:

(7) $$F^{\left(l\right)}=\{\begin{array}{ll} 0, & h\geq h_{c},\\ -1.5\pi \rho_{f}\nu {\left[D_{p}\left(\frac{1}{h}-\frac{1}{h_{c}}\right)\right]}^{1.5}U_{p}, & h<h_{c}, \end{array}$$

where $\nu =c_{s}^{2}\left(\tau -0.5\right)\Delta t$ is the kinematic viscosity of the fluid; $D_{p}$ is the diameter of the particle; $U_{p}$ is the particle velocity towards the wall; h is the minimum gap between the particle and the wall; $h_{c}=1.5\Delta x$ is the cutoff distance whether to consider the lubrication force or not. 

By summation of the forces and torques acting on the particle, the movement of the particle can be solved explicitly using Newton’s second law:

(8) $$\begin{array}{l} a_{p}^{t+\Delta t}=\left(\sum^{\;}F^{\left(h\right)}+\sum^{\;}F^{\left(c\right)}+\sum^{\;}F^{\left(u\right)}+F^{\left(l\right)}\right)/m_{p},\\ u_{p}^{t+\Delta t}=u_{p}^{t}+0.5\left(a_{p}^{t+\Delta t}+a_{p}^{t}\right)\Delta t,\\ x_{p}^{t+\Delta t}=x_{p}^{t}+0.5\left(u_{p}^{t+\Delta t}+u_{p}^{t}\right)\Delta t,\\ \alpha_{p}^{t+\Delta t}=\left(\sum^{\;}T^{\left(h\right)}+\sum^{\;}T^{\left(c\right)}+\sum^{\;}T^{\left(u\right)}\right)/I_{p},\\ w_{p}^{t+\Delta t}=w_{p}^{t}+0.5\left(\alpha_{p}^{t+\Delta t}+\alpha_{p}^{t}\right)\Delta t,\\ \theta_{p}^{t+\Delta t}=\theta_{p}^{t}+0.5\left(w_{p}^{t+\Delta t}+w_{p}^{t}\right)\Delta t, \end{array}$$

where $a_{p}$, $u_{p}$, $\alpha_{p}$, $w_{p}$, $\theta_{p}$, $m_{p}$, and $I_{p}$ are the translational acceleration, velocity, rotational acceleration, rotational velocity, angle, mass and moment inertia of the particle.

### 2.4. Problem

**Figure 3:**
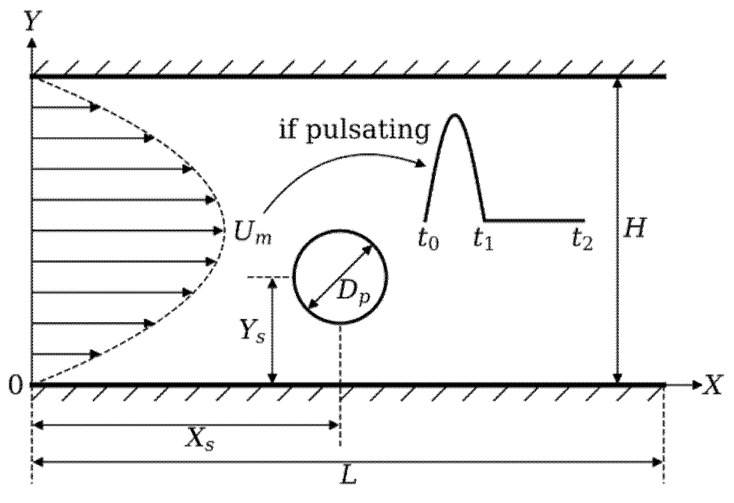
Configuration of a particle migrating in Poiseuille channel flow. The origin coordinate locates at left down corner, X in horizontal direction and Y in vertical direction. The simulation domain size is fixed as L×H, while the particle is located at $\left[X_{s},\;Y_{s}\right]$ initially. The diameter of the particle is $D_{p}$, $U_{m}$ represents the maximum velocity. For pulsatile case, $U_{m}$ will change by time.

The configuration of one circular particle migrating in Poiseuille flow is shown in Figure 3. A parabolic velocity profile with the maximum velocity $U_{m}$ is set at the left inlet boundary in the positive X direction, and the velocity in Y direction is zero. For $U_{m}$, there are two cases: non-pulsatile or pulsatile. If pulsatile, $U_{m}$ will change over time, otherwise, it will be a constant. For considering reproducibility of this work, patient-specific velocity profile [50] will not be adopted to impose at the inlet boundary. The half-period of the sinusoidal function at time interval $\left[t_{0},\;t_{1}\right]$ (systolic period) and zero at $\left[t_{1},\;t_{2}\right]$ (diastolic period) is utilized which can be seen from Figure 3. In the systolic period, $t_{1}-t_{0}=0.3\;s$, and in the diastolic period, $t_{2}-t_{1}=0.5\;s$, which means one cardiac cycle lasts 0.8 s. The unit conversion factor from lattice time to physical time is $10^{-5}$. At the right boundary, the normal derivative of the velocity is zero and the pressure is set to be $p_{out}=\rho_{f}c_{s}^{2}\;$ [19]. No-slip boundary conditions are imposed at the top and bottom channel walls.

If there are no additional statements, the channel length L is 500Δx and the height H is 100Δx. TEP is basically unchanged for different channel length (300Δx, 400Δx, 500Δx, 600Δx, 700Δx and 2000Δx). So L=500Δx is adopted same as Wen et al. [17]. As shown in Figure 3, the circular particle center is located at $\left[X_{s},\;Y_{s}\right]$ initially. The diameter of the circular particle in this work is 15Δx, 20Δx, 25Δx, 30Δx, 35Δx, and 40Δx, which means $k=D_{p}/H=0.15,\;0.20,\;0.25,\;0.30,\;0.35,\;0.40$, respectively. Meanwhile, $Re=U_{m}H/\nu =25,\;50,\;75,\;100,\;150,\;200$ is studied by changing the kinematic viscosity of the fluid.

Driven by the fluid velocity, the particle will always travel along X axis in positive direction, so an infinite channel is needed to avoid the particle moving out of the simulation domain which can be achieved by moving domain technique [6,51,52]. When the X-coordinate of the particle exceeds $X_{s}+\Delta x$, the fluid field and the particle need to shift on lattice spacing left, which ensures that the particle will never travel too far from its original position. Meanwhile, it should be noted that the actual moving distance along X axis in positive direction should add up one lattice spacing when performing this shift once.

## 3. Validation

To validate the accuracy of our LBM code, two benchmark cases are implemented below.

**Figure 4:**
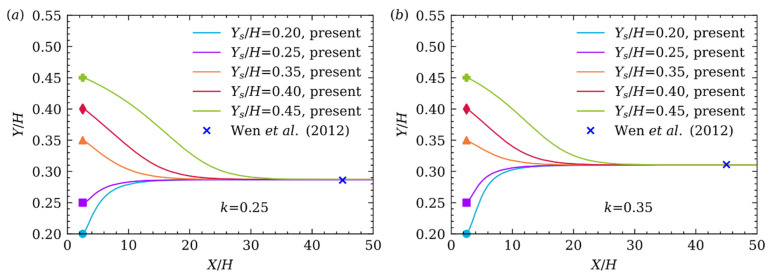
Lateral migration of the particle released from different initial positions in Poiseuille flow with (a) k=0.25, (b) k=0.35.

In the first case, Re is set to be 50, and the terminal particle Reynolds number $Re_{p}=U_{x,p}D_{p}/\nu$ is about 9.63, where $U_{x,p}$ is the terminal particle velocity in X direction. It is very close to $Re_{p}$ while the particle is driven by pressure difference [17]. Our simulating results with k=0.25 and k=0.35 are plotted in Figure 4a,b, respectively. The SS effect is quite obviously found and TEP of the particle has nothing to do with the initial horizontal position ($Y_{s}/H=0.20,\;0.25,\;0.35,\;0.40,\;0.45$) which only changes the trajectory from initial position to TEP. All the results, even the curve shape shown in Figure 4a,b are consistent with those of Wen et al. [17].

**Figure 5:**
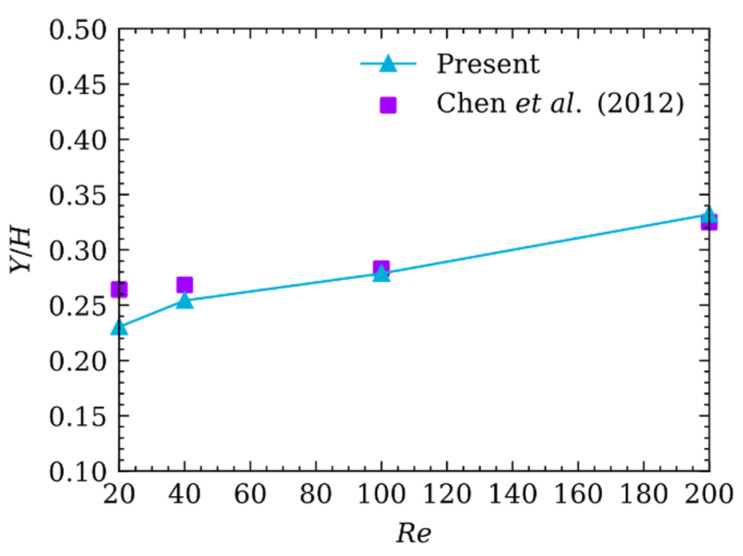
Comparison of TEPs of the particle migrating in Poiseuille flow at different Re.

The foregoing results are validated only when Re is 50. The second case is adopted to validate over the entire range of Re=20, 40, 100, 200. The particle diameter is $D_{p}=22$, the channel width is H=200, and the channel length is L=1000, which leads to the blockage ratio being k=0.11. Figure 5 shows the comparison of the present results with previous ones simulated by Di Chen et al. [53] The comparison shows TEPs are in good agreement and the particle will be closer to channel centerline with increasing Re in 2D Poiseuille flow.

## 4. Results and Discussions

### 4.1. Fluid and Particle Interaction

**Figure 6:**
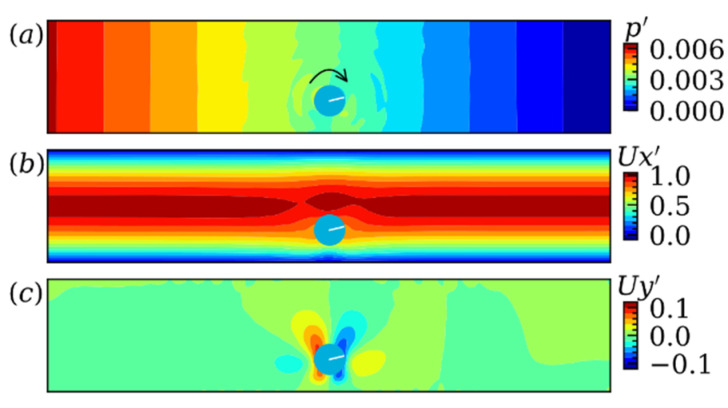
The contour view of (a) the dimensionless pressure $p^{\prime }$, (b) the dimensionless fluid velocity in X direction $U_{x}^{\prime }$ and (c) in Y direction $U_{y}^{\prime }$ in stable state of particle migrating in non-pulsatile flow at k=0.25 and Re=50.

After validation, we first simulate the particle migrating in non-pulsatile flow at k=0.25 and Re=50. Figure 6a–c, shows the contour view of the dimensionless pressure $p^{\prime }=\left(p-p_{out}\right)/p_{out}$, the dimensionless fluid velocity in X direction $U_{x}^{\prime }=U_{x}/U_{m}$ and in Y direction $U_{y}^{\prime }=U_{y}/U_{m}$, respectively. Due to the moving domain method adopted here, the particle will always locate around the middle of the simulation domain which is significantly disturbed by the present of the particle. Obviously, the pressure strip can be observed upstream and downstream of the particle. The pressure at the upper left and down right side of the particle is higher than the upper right and down left corner, and hence generates a particle rotation in clockwise direction illustrated by the black arrow in Figure 6a. Due to non-slip boundary condition at the boundary location of particle, clockwise rotation of the particle will induce fluid flowing upward at left and downward at right as shown in Figure 6c. Moreover, it can be seen from Figure 6b that the particle follows with fluid movement quite well [54].

### 4.2. Trajectory

Several dominant forces acting on the particle drive it to TEP in Y direction. The first force is shear gradient lift force due to the parabolic velocity profile, which points to the wall. The second force is wall induced lift force due to the interaction between the particle and the channel wall calculated by using added lubrication force model introduced before which directs towards the channel centerline. The third force is called Magnus force [55] due to the rotation of the particle migrating in fluid. As demonstrated above, the particle in non-pulsatile flow travels in X direction and at the same time it rolls in clockwise direction. Thus, the Magnus force is directed to the channel centerline. Certainly, when the particle moves in fluid, it will be affected by the drag force. In summary, there are at least four forces governing particle migration in fluid suspension.

**Figure 7:**
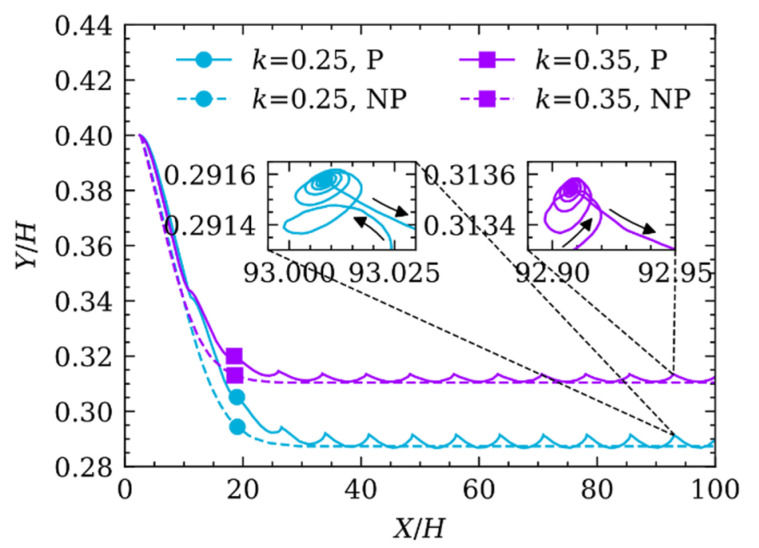
The particle center trajectory for k=0.25 (two sky-blue curves with circle marker), k=0.35 (two purple curves with square marker) at Re=50. Solid curve represents the case driven by pulsatile velocity, which is abbreviated as P, while NP is the acronym for non-pulsatile flow donated by dash curve.

Figure 7 shows the particle center trajectory along the channel for k=0.25 and k=0.35 at Re=50. Obviously, several cardiac cycles later, the particle driven by pulsatile velocity migrates around TEP in non-pulsatile flow periodically. In the systolic period, the particle laterally migrates towards wall mainly affected by shear gradient lift force. While in the diastolic period, the shear gradient lift force disappears, and the particle still rotates because of inertia, so it will migrate back to the channel centerline under the Magnus force. The moving direction is illustrated by black arrows which are shown in the subplots of Figure 7. The particle oscillates in spiral-shaped structures and will travel towards the wall again in another systolic period.

**Figure 8:**
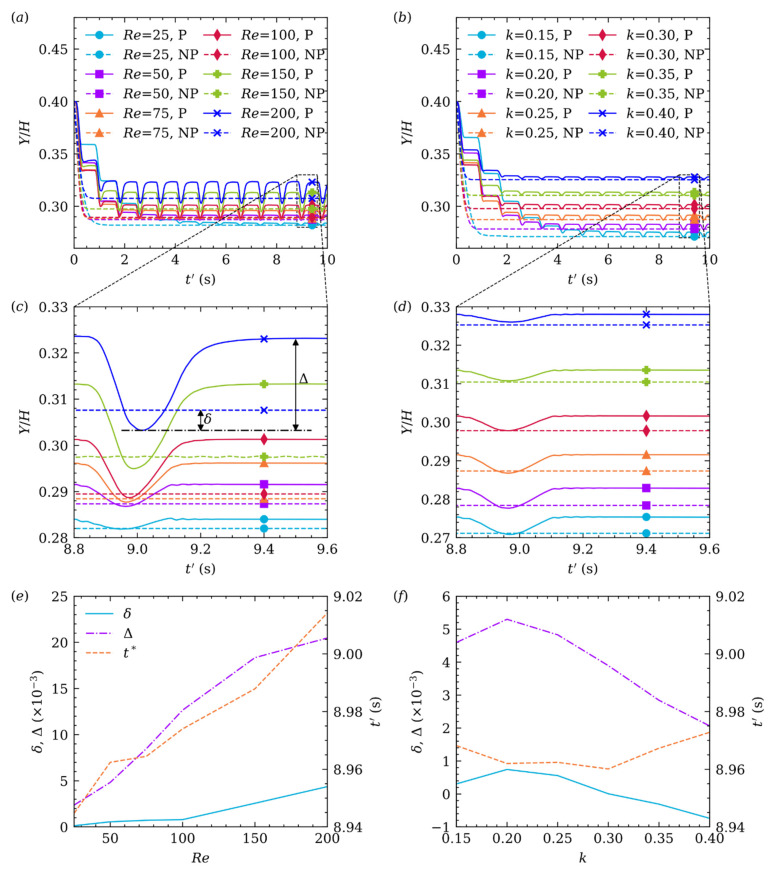
Time history of the particle center trajectory at different Re (a,c) at k=0.25, and different k (b,d) at Re=50. The variation of $\delta ,∆,t^{*}$ with Re (e) and k (f).

Figure 8a,c gives the particle center trajectory versus time $\left(t^{\prime }=t/10^{5}\right)$ variation with Re at k=0.25, and (b,d) donates different k at Re=50. Moreover, (c,d) is the enlarged view of (a,b) in one stable cardiac cycle $\left(t^{\prime }=\left[8.8,\;9.6\right]\right)$ illustrated by black dash line box, respectively. Overall, TEPs are all closer to the channel centerline when Re or k increases, whether or not in pulsatile flow. Consequently, the particle will take longer (or more cardiac cycles) to reach TEP. As shown in Figure 8c, we define ∆ as the dimensionless distance between the highest and lowest position in this stable cardiac cycle, δ is the signed dimensionless distance between TEP in non-pulsatile flow and the lowest position (negative value means the particle did not exceed TEP in non-pulsatile flow), and $t^{*}$ is time in this cardiac cycle when the particle locates the lowest position. In general, the influence of Re on $\delta ,∆,t^{*}$ is greater than k. As Re increases, the viscosity of the fluid decreases, thus the drag force acts on the particle decreases. The particle, therefore, can laterally migrate farther in systolic period, even exceeds TEP in non-pulsatile flow. Meanwhile, the particle will take longer to migrate from the highest position to the lowest position for its longer travel distance. As a result, Figure 8e shows that δ, ∆ and $t^{*}$ all increase monotonously with increasing Re. The effect of k on $\delta ,∆,t^{*}$ is a little more complicated. As k increases, the particle inertia increases, and the drag force also increases. Shear gradient force increases with increasing k, but decreases when the particle is closer to channel center. Consequently, it can be seen from Figure 8f, δ, ∆ increases at first and then decreases when k increases, the maximum of δ, ∆ occurs at k=0.2. Moreover, $t^{*}$ was mostly unchanged with increasing k.

### 4.3. Orientation

**Figure 9:**
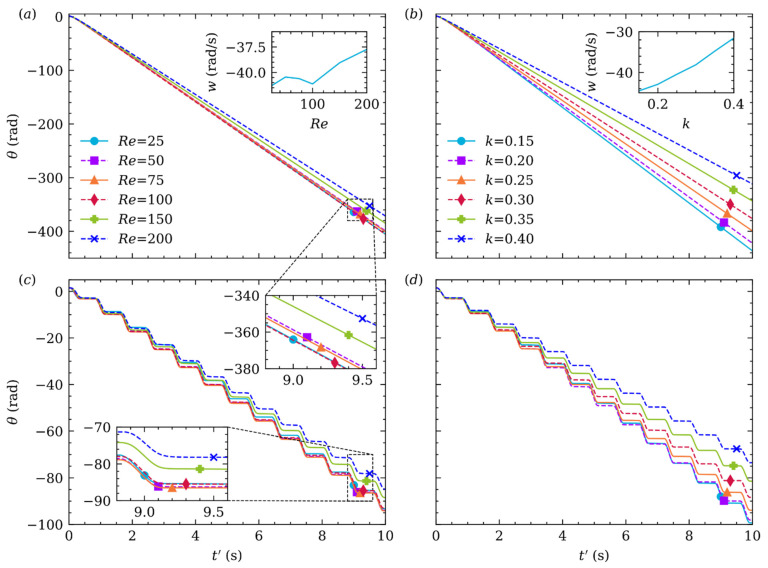
The particle orientation at different Re (a,c) and k (b,d). Meanwhile, (a,b) is in non-pulsatile and (c,d) in pulsatile flow.

Observed in Figure 9, the particle always rotates clockwise while migrating in the channel, irrespective of Re, k, and whether or not in pulsatile flow. In the diastolic period, the particle rotates much slower but still in the clockwise direction. If the particle is closer to the channel wall, it will experience larger gradient of fluid velocity. As a result, the smaller Re or k is, the faster the particle rotates. Figure 8 shows that, when Re<100, TEP of the particle is very similar, so they rotate almost at the same speed (w) which is shown in the subplot of Figure 9a. In addition, it can be seen in the subplot of Figure 9b, the particle rotate speed is almost linearly coherent with k. Furthermore, the moment inertia of the particle is proportional to the square of the particle diameter, i.e., k. Consequently, by comparing Figure 9a,b, the influence of k on w is much larger than Re.

### 4.4. Damping

As mentioned before, the particle oscillates in the diastolic period which is shown in Figure 7. We choose the particle velocity which indicates the interaction between the particle and fluid [56] for analysis. Moreover, the particle velocity in X direction $\left(U_{x}\right)$ is preferred, because the particle velocity curve is not center symmetry in Y direction.

**Figure 10:**
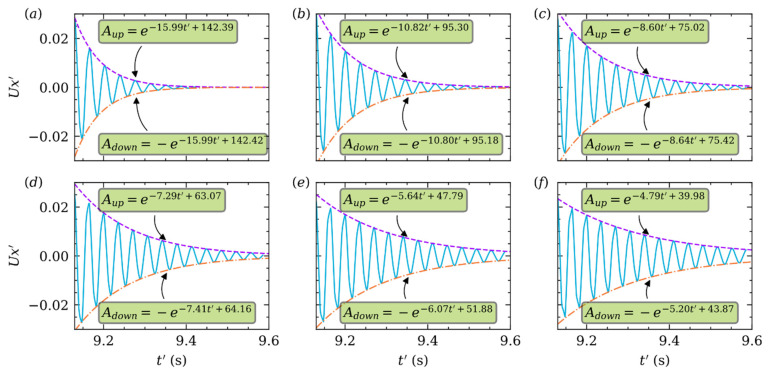
Damping of $U_{x}^{\prime }$ (solid line in sky-blue color) of the particle at k=0.25 and different Re=(a) 25, (b) 50, (c) 75, (d) 100, (e) 150, (f) 200. The upside (dashed line in purple color) and downside (dash dotted line in orange color) envelope curves are fitted by exponential function.

**Figure 11:**
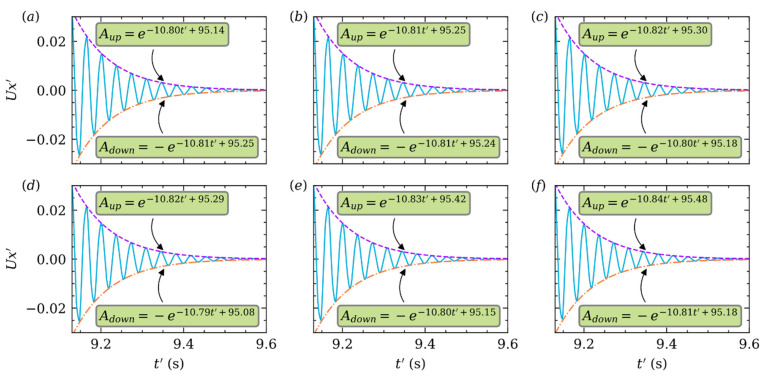
Damping of $U_{x}^{\prime }$ at Re=50 and different =(a) 0.15, (b) 0.20, (c) 0.25, (d) 0.30, (e) 0.35, (f) 0.40. Envelope curves are fitted in the same way.

For better illustration, the particle velocity in X direction is normalized by: $U_{x}^{\prime }=U_{x}/U_{m}$. The curves of $U_{x}^{\prime }$ in Figure 10 are much like a spring-mass system which is underdamped. Figure 10a shows, when Re is small, $U_{x}^{\prime }$ damps out rapidly after several quasi periods. If Re is small enough, like Re≈1, over damped is expected. Obviously, in Figure 10a–f, the damping effect becomes weaker with increasing Re. We fit the upside and downside envelope curve by using exponential function for purpose. For example, $A_{up}=e^{-15.99t^{\prime }+142.39}$ in Figure 10a, and the decay rate λ=15.99. The constants of fitting exponential function of the upside and downside are almost the same for each Re. And the absolute values of those constants decrease with increasing Re. The influence of k is also analyzed, but it can be seen from Figure 11a–f that k has almost no effect on damping.

**Figure 12:**
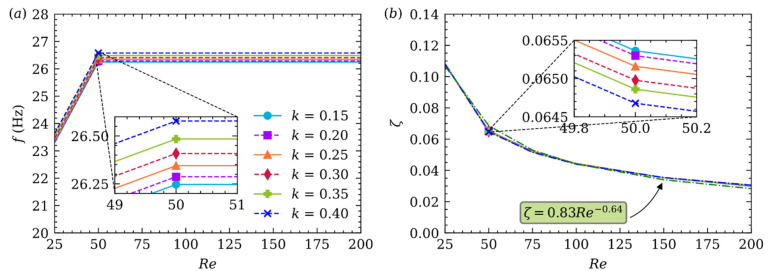
The quasi frequency (a) and damping ratio (b) at different Re and k.

Figure 12a gives the quasi frequency ($f_{q}$) of $U_{x}^{\prime }$ oscillation at different Re and k. It can be seen from the subplot that the quasi frequency increases as increasing k, but the impact of k on the quasi frequency is relatively small. However, the quasi frequency will not change when Re increases except for Re=25. As mentioned earlier, in Figure 10a, when Re=25, $U_{x}^{\prime }$ damps out rapidly after several quasi periods. The first few quasi periods are longer than the last ones, which will result in smaller quasi frequency when Re=25.

As shown in Figure 12b, the damping ratio $\zeta \approx \lambda /\left(2\pi f_{q}\right)$, in our cases, is always smaller than 1, which determines this is an underdamped system, and this system will die out slower when ζ decreases. Additionally, ζ of $U_{x}^{\prime }$ oscillation basically does not change with variation of k, but it decreases with increasing Re, i.e., Re is the decisive parameter on ζ [57]. A similar conclusion is made by C.A. Coulomb in 1784 by using several material plates hanging by a metal wire with an initial torsion angle and then released to start timing until the plate oscillation until dying out. ζ is only related to the viscosity of fluid and not the material of plate. The reason for damping is the friction inside the fluid which is called Newton’s law of friction, not the interaction friction between the plate and the fluid. Finally, in Figure 12b, the relation between ζ and Re is fitted as: $\zeta =0.83Re^{-0.64}$.

## 5. Conclusions

IB-LBM was used to simulate one neutrally buoyant circular particle migration in 2D Poiseuille channel flow driven by pulsatile and non-pulsatile velocity. The moving domain technique is adopted to achieve that the particle can migrate in infinite channel. The results show that the particle moving in pulsatile flow is slightly different from that in non-pulsatile one. It will laterally migrate back to the channel centerline with small oscillations in the spiral-shaped structure during the diastole and move back toward TEP during the systole. The effect of Re on ζ is decisive. This research may shed some light on understanding the particle behavior in Poiseuille flow driven by pulsatile velocity for optimizing therapeutic nanoparticles system in arteries.

The limitation of this work is that Re varies only from 25 to 200. Smaller Re leads to bigger SRT, thus the simulation will not be accurate. In contrast, bigger Re results in smaller SRT which the simulation will diverge. Furthermore, k range only from 0.15 to 0.40. Because the channel height is set to be 100 lattice units, if k is smaller than 0.15, the simulation resolution will not be adequate. On the other hand, if k is bigger than 0.4, half channel width will be blocked by the particle. Parameters need to choose carefully to get a more varied range of Re and k. This will be a future direction of this work. Another future direction will be considering more particles or simulating particle migration in three-dimensional pipe pulsatile flow.

## Data Availability

The data that support the findings of this study are available from the first author upon reasonable request.
